# A Novel Method of Human Joint Prediction in an Occlusion Scene by Using Low-Cost Motion Capture Technique

**DOI:** 10.3390/s20041119

**Published:** 2020-02-18

**Authors:** Jianwei Niu, Xiai Wang, Dan Wang, Linghua Ran

**Affiliations:** 1 School of Mechanical Engineering, University of Science and Technology Beijing, Beijing 100083, China; wangxiai_wang@163.com (X.W.); 13426023594@163.com (D.W.); 2 China National Institute of Standardization, Beijing 100191, China; ranlh@cnis.ac.cn

**Keywords:** Kinect, Kalman filter, occlusion, uman joint prediction

## Abstract

Microsoft Kinect, a low-cost motion capture device, has huge potential in applications that require machine vision, such as human-robot interactions, home-based rehabilitation and clinical assessments. The Kinect sensor can track 25 key three-dimensional (3D) “skeleton” joints on the human body at 30 frames per second, and the skeleton data often have acceptable accuracy. However, the skeleton data obtained from the sensor sometimes exhibit a high level of jitter due to noise and estimation error. This jitter is worse when there is occlusion or a subject moves slightly out of the field of view of the sensor for a short period of time. Therefore, this paper proposed a novel approach to simultaneously handle the noise and error in the skeleton data derived from Kinect. Initially, we adopted classification processing to divide the skeleton data into noise data and erroneous data. Furthermore, we used a Kalman filter to smooth the noise data and correct erroneous data. We performed an occlusion experiment to prove the effectiveness of our algorithm. The proposed method outperforms existing techniques, such as the moving mean filter and traditional Kalman filter. The experimental results show an improvement of accuracy of at least 58.7%, 47.5% and 22.5% compared to the original Kinect data, moving mean filter and traditional Kalman filter, respectively. Our method provides a new perspective for Kinect data processing and a solid data foundation for subsequent research that utilizes Kinect.

## 1. Introduction

The development of robotics technology is driving the application of robots from industrial production to the military, medical, and service fields [1,2,3]. In industrial production lines, industrial robots can replace workers in various tasks, such as assembly, handling, pick-up and welding, which can greatly improve work efficiency [4]. In the military, robots can be operated to perform dangerous tasks, such as bomb and mine defusing [5]. However, in the service field, robots are often used to handle more complex tasks that require people’s involvement [6]. Therefore, the combination of robot control technology and human-computer interaction technology can effectively improve the working ability and intelligence of civil robots [7,8]. 

At present, the control of civilian robots has been transformed from the traditional manual control mode, such as remote control and operation handling, to the vision-based robot control mode [9]. Visual-based robotic somatosensory control methods are gaining increasing applications, such as the treatment of children with autism, robot classroom teaching, and assisting robots [10,11,12]. This type of control mode is simple to operate, more in line with the human mindset and easy to perform by even children and elderly people. However, how to obtain human motion information as a control signal for somatosensory operation of the robot is an urgent problem that needs to be solved. Marker-based motion capture settings (such as VICON, https://www.vicon.com) are a potential solution in this area because of their proven accuracy [13,14], but they are very expensive and cumbersome to use. Robot control based on color image information is the current main somatosensory robot control method [15]. This control method is simple, natural and convenient, but it is subject to environmental lighting, background complexity and human skin color. Thus, an inexpensive, environmentally unaffected system is essential for robot control.

Kinect is a somatosensory sensor from Microsoft that is low-cost and mainly used in the civilian field [16]. Kinect uses an infrared (IR) projector, an IR sensor and an RGB (Red Green Blue) camera to track human joints in three-dimensional (3D) space, which enables it to analyze joint kinematics [17,18,19]. However, the skeleton data obtained from the Kinect exhibit a high level of jitter due to noise and estimation error. This jitter worsens when there is occlusion or a subject moves slightly out of the field of view of the sensor for a short period of time [20]. Nonetheless, researchers have shown great interest in Kinect and applied it to home-based rehabilitation, clinical assessments and ergonomics. Wochatz et al. [21] think that the Kinect system can reliably assess lower limb joint angles and positions during simple rehabilitation exercises. Sarsfield et al. [22] present a clinical qualitative and quantitative analysis of the pose estimation algorithms of Kinect to assess its suitability for technology-supervised rehabilitation and to guide the development of future pose estimation algorithms for rehabilitation applications. Manghisi et al. [23], Xu et al. [24] and Plantard et al. [25] suggest a Rapid Upper Limb Assessment (RULA) assessment using the Kinect v2 sensor, where an ergonomic assessment is performed by computer processing and skeleton tracking. However, if the data obtained by Kinect are inaccurate, it will seriously affect these studies, thus, it is necessary to process Kinect skeleton data.

Various approaches are employed to stabilize joint coordinates. The main approaches are filter algorithms, such as the amplitude-limited filter, moving mean filter and Kalman filter. Edwards and Green [26] compared four different filter-based approaches to obtain smooth joint coordinates: the Kinect SDK’s built-in Holt double exponential smoothing filter, an averaging filter, a Kalman filter with a constant-value model, and a Kalman filter with a Wiener Process Acceleration (WPA) model. Du and Zhang [27] proposed an innovative amplitude-limited algorithm of over-damping to solve the problem of error extraction and dithering due to the noncontact measure. Rosado et al. [28] improved the accuracy of the motions captured by Kinect from both static and dynamic aspects. Static calibration was used to obtain the average static distance of adjacent joints, and the joint position was optimized in the dynamic calibration using this static distance. Wang et al. [29] proposed a kinematic filtering algorithm based on the Unscented Kalman Filter and kinematic model of the human skeleton. The proposed algorithm can obtain a smooth kinematic parameter with reduced noise compared to the kinematic parameter generated from the raw motion data from Kinect. The traditional time series filter method has real-time performance and low algorithm complexity, which can partially remove jitter and noise from the Kinect joint data. However, abnormal joint data with large errors cannot be completely eliminated. 

Researchers have adopted various approaches for dealing with abnormal joint data with large errors, for example, the joint estimation algorithm. Shen et al. [30] proposed an exemplar-based method to learn to correct the initially estimated joint-based skeleton, and observed a significant improvement compared to the approaches delivered by the current Kinect system. Shum et al. [31] proposed a set of erroneous data identification methods and established a human joint posture database to find the best substitute data from erroneous data. Liu et al. [32] proposed a posture reconstruction method based on a local mixture of Gaussian process models that Plantard et al. [33] adopted to filter pose graphs for efficient Kinect pose reconstruction. Approaches for abnormal joint data with large errors mostly use the real joint as a reference to learn the relationship between Kinect joint data and real joint data through machine learning, thus, they have high complexity and require real joint data as a reference. Moreover, different models have been developed for different types of motion learning, so they are not suitable for practical applications.

In the present paper, the advantages of the two methods are combined. We proposed a reliability index to identify abnormal joint data with large errors. Then, we improved the traditional Kalman filter according to various human movement constraints to realize the low-complexity joint correction algorithm. In the rest of the paper, Section 2 describes the proposed method. In Section 3, the experimental setup is explained, which also includes the experiment results. The conclusions and scope of future work are discussed in Section 4.

## 2. Methodology

### 2.1. Reliability Measurement

An incorrect skeleton joint in a motion capture system is even more damaging than a missed joint since it incorrectly guides the system to infer posture. Therefore, we applied an index called the vibration degree to evaluate the reliability of the joint [28].

When Kinect cannot accurately track a joint, there is a high-frequency vibration of the joint. Assuming $p_{i}(f)=(x_{1},y_{1},z_{1})$ and $p_{i}(f+1)=(x_{2},y_{2},z_{2})$ to be the 3D position of skeleton *i* in two successive frames, we can calculate the displacement vectors as:

(1) $$d_{i}(f)=p_{i}(f+1)-p_{i}(f)=(x_{2}-x_{2},y_{2}-y_{1},z_{2}-z_{1})$$

The angle between continuous displacement vectors can be described as:

(2) $$\theta_{i}(f)=\{\begin{array}{cc} \arccos(\frac{d_{i}(f)•d_{i}(f+1)}{\left|d_{i}(f)\right|\left|d_{i}(f+1)\right|}) & if\left|d_{i}(f)\right|>d_{\min},\left|d_{i}(f+1)\right|>d_{\min}\\ 0 & otherwise \end{array}$$

where $d_{\min}$ is the minimum distance value of an acceptable displacement vector. $d_{\min}$ is used to avoid a large change in angle caused by small changes when the joint position is basically stationary. In our experiment, the distance value of a displacement vector is approximately 0.01 m when the joint position is basically stable. By contrast, when the joint position is unstable, the distance of the displacement vector increases. Therefore, the $d_{\min}$ value is set to 0.02 m in our experiment. 

The vibration degree reliability is defined as:

(3) $$R_{i}(f)=1-\frac{\max(\min(\theta_{i}(f),\theta_{\max})-\theta_{\min},0)}{\theta_{\max}-\theta_{\min}}$$

where $\theta_{\max}$ and $\theta_{\min}$ are the extremities of human body movement. $\theta_{\min}$ is the lower limit of the angle change when there is jitter between each frame, and $\theta_{\max}$ is the upper limit of the angle change that we consider. Based on Morasso [34], which is concerned with kinesiology, we set $\theta_{\min}$=45° and $\theta_{\max}$=135°. However, the setting of the threshold values here are empirically determined, and this limitation is expected be overcome in our future research.

### 2.2. Reliability Threshold

The main advantages of Kinect sensors are their low price, ease of use and adaptability to the environment. However, all sensors produce measurement errors and noise when measuring physical quantities. The Kinect sensor is an inaccurate system that provides joint measurement data with certain measurement errors and noise [35]. These errors and noise are generated by various factors, which can be classified into two main types. The first type is the lack of joint position information caused by occlusion and the part of the human body that leaves the measurement range. The Kinect sensor estimates the missing joint using the estimation algorithm and can obtain erroneous data. The second type is the systematic error introduced by quantization noise and sensor stability. The first type of data error may cause joint data to significantly deviate from the true value, which affects the accuracy of the joint data; the second type of error has a small amplitude but appears more frequently, which results in uneven joint data [36]. Therefore, this paper classifies the two types of joint data and performs the corresponding processing after classification. We applied the vibration degree introduced in Section 2.1 to evaluate the reliability of the joint and determine the reliability threshold to divide the two types of joint data. Joint data with lower reliability than the threshold are recognized as abnormal data and are called erroneous data, and data with higher reliability than the threshold are identified as data to be optimized and are called noise data. This paper used the common approximation method in mathematics to obtain the joint reliability threshold, as follows.

**Figure 1:**
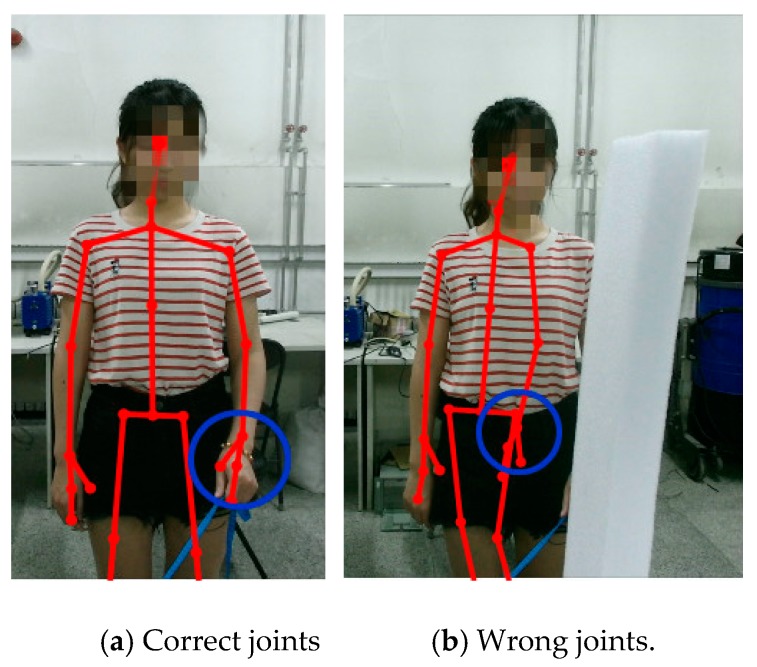
Visualization of skeleton tracking.

First, the occlusion marker was artificially set in the experimental scene. Second, we obtained the motion data through occlusion from the Kinect and calculated the joint reliability. Third, we simultaneously collected the human motion color image information to manually mark wrong joint data frame by frame, as shown in Figure 1. Finally, we used the approximation idea to determine the joint reliability threshold. When the current threshold identification error data are lower than the manual labeling, the current threshold is set to the lower threshold. When the current threshold identification error data are higher than the manual labeling, the current threshold is set to the upper threshold. The approximation algorithm stops when the threshold judgment and manual labeling error are within 10 percent of each other.

In the present paper, wrist joint motion data of five subjects were collected. Each subject repeated five experiments, and each experiment collected 150 frames of data. We manually marked the number of frames of wrong joint and used the approximation algorithm to determine the reliability threshold. The results are shown in Table 1. Generally speaking, the data difference is not big enough, which may lead to doubts about the rationality of classification. However, in our opinion, the difference is a relative concept. Whether the difference is significant or not depends on the specific application. For example, if the proposed method in this paper is applied in the simulation of physical exercise such as table tennis playing, the data difference we provided is not big enough since the amplitude of the arm of the player in such kind of motion is quite big. In contrast, if the proposed method in this paper is applied in the simulation of rehabilitation training of patients with Parkinson’s, the data difference we provided is very big since the amplitude of the arm of the patients in such kind of motion is quite small. Therefore, we determined the reliability threshold is the average value of the experimental data from 25 groups of 0.70 based on the results in Table 1 eventually. We defined erroneous data as joint data with a reliability threshold below 0.70 and noise data as joint data with a reliability threshold above 0.70. 

### 2.3. Algorithm to Handle Noise Data

Joint data with a reliability threshold above 0.70 are defined as noise data, and a Kalman filter is used to smooth the noise of the data. Except for separately obtaining each joint coordinate, we used Kinect to collect the sound source angle of the subject. Therefore, the state vector is taken to be the true 3D coordinates of the skeleton joint and their velocities and is written as $X={\left[x,y,z,\dot{x},\dot{y},\dot{z}\right]}^{T}$. The measurement vector is taken to be the true 3D coordinates of the skeleton joint and sound source angle and is written as $Y={\left[x,y,z,\arctan(x/z)\right]}^{T}$. The state transition process is modeled as a linear dynamic system, and the measurement is modeled as a nonlinear dynamic system, where the next state at time instance *k*+1 is expressed in terms of the previous state at the *k*th instance and mathematically represented as:

(4) $$X_{k+1}=FX_{k}+Q_{k}$$

(5) $$Y_{k}=h(X_{k})+R_{k}$$

where $X_{k}$ and $Y_{k}$ are the state vector and measurement vector, respectively, at time instant *k*; $Q_{k}$ and $R_{k}$ are the process noise and measurement noise, respectively; *F* is the state transition matrix; and h is the state transformation function. 

Matrix *F* is given in block form by:

(6) $$F=\left(\begin{array}{cccccc} 1 & 0 & 0 & T & 0 & 0\\ 0 & 1 & 0 & 0 & T & 0\\ 0 & 0 & 1 & 0 & 0 & T\\ 0 & 0 & 0 & 1 & 0 & 0\\ 0 & 0 & 0 & 0 & 1 & 0\\ 0 & 0 & 0 & 0 & 0 & 1 \end{array}\right)$$

For state transformation function h, we adopted the extended Kalman Filter to linearize *h* and replace matrix *H* in the filter with the Jacobian of *h*, which is evaluated at the current state estimate as:

(7) $$H_{k}=\left(\begin{array}{cccccc} 1 & 0 & 0 & 0 & 0 & 0\\ 0 & 1 & 0 & 0 & 0 & 0\\ 0 & 0 & 1 & 0 & 0 & 0\\ \frac{1/{\hat{z}}_{k}^{-}}{1+{\left({\hat{x}}_{k}^{-}/{\hat{z}}_{k}^{-}\right)}^{2}} & 0 & \frac{{\hat{x}}_{k}^{-}/{\left({\hat{z}}_{k}^{-}\right)}^{2}}{1+{\left({\hat{x}}_{k}^{-}/{\hat{z}}_{k}^{-}\right)}^{2}} & 0 & 0 & 0 \end{array}\right)$$

Kalman filter estimates ${\hat{X}}_{k}$ from $X_{k}$ with the knowledge of measurement vector $Y_{k}$ in two steps: prediction and update. The standard Kalman filtering prediction step can be written as:

(8) $${\hat{X}}_{k}^{-}=F{\hat{X}}_{k-1}$$

(9) $$P_{k}^{-}=FP_{k-1}^{-}F^{T}+Q_{k}$$

where $P_{k}^{-}$ is the covariance matrix associate with prediction ${\hat{X}}_{k}^{-}$ for an unknown true state $X_{k}$ and is expressed as:

(10) $$P_{k}^{-}=E[(X_{k}-{\hat{X}}_{k}^{-}){\left(X_{k}-{\hat{X}}_{k}^{-}\right)}^{T}]$$

The updated state based on the measurement is expressed as:

(11) $$K_{k}=P_{k}^{-}H^{T}{\left(HP_{k}^{-}H^{T}+R\right)}^{-1}$$

(12) $${\hat{X}}_{k}={\hat{X}}_{k}^{-}+K_{k}(Y_{k}-H{\hat{X}}_{k}^{-})$$

(13) $$P_{k}=(I-K_{k}H)P_{k}^{-}$$

where $K_{k}$ is the Kalman gain matrix. The Kalman filter minimizes the mean square error between the estimated ${\hat{X}}_{k}$ and true $X_{k}$, providing smoother coordinates.

### 2.4. Algorithm to Handle Erroneous Data

**Figure 2:**
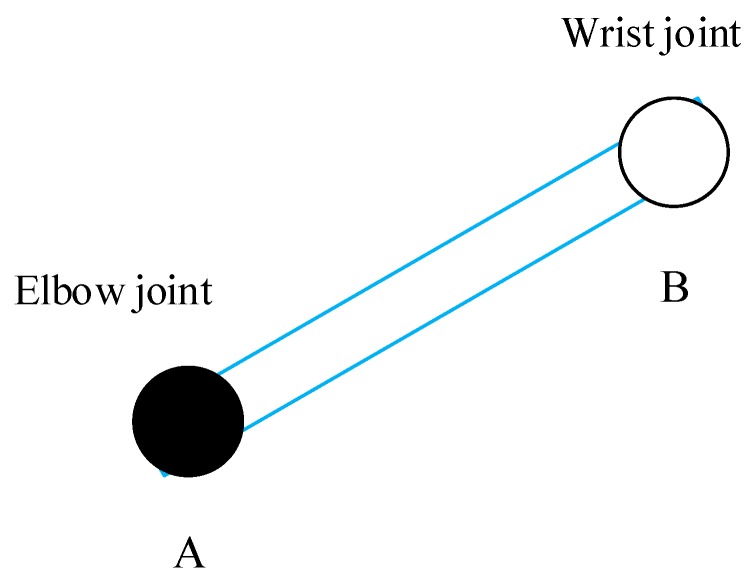
Schematic diagram of the error joint and its parent joint.

We define erroneous data as joint data with a reliability threshold below 0.70, and a Kalman filter with human model constraints is used to correct the error of the data. To illustrate the algorithm to handle erroneous data, we assume that the wrong joint is wrist joint B at the *k*th frame and that its parent joint is elbow joint A $\left(X_{1},Y_{1},Z_{1}\right)$, as shown in Figure 2.

First, the Kalman filter algorithm was used to estimate the motion trend between frames to obtain the error joint position estimate $P(\tilde{X},\tilde{Y},\tilde{Z})$. Then, we established the constraint equation. Since the length of the human skeleton is constant, it is estimated that the error joint should be on the spherical surface with radius $l_{AB}$ at the center of the parent node. The constraint equation is as follows:

(14) $${\left(X-X_{1}\right)}^{2}+(Y-Y_{1})+(Z-Z_{1})={l_{AB}}^{2}$$

**Figure 3:**
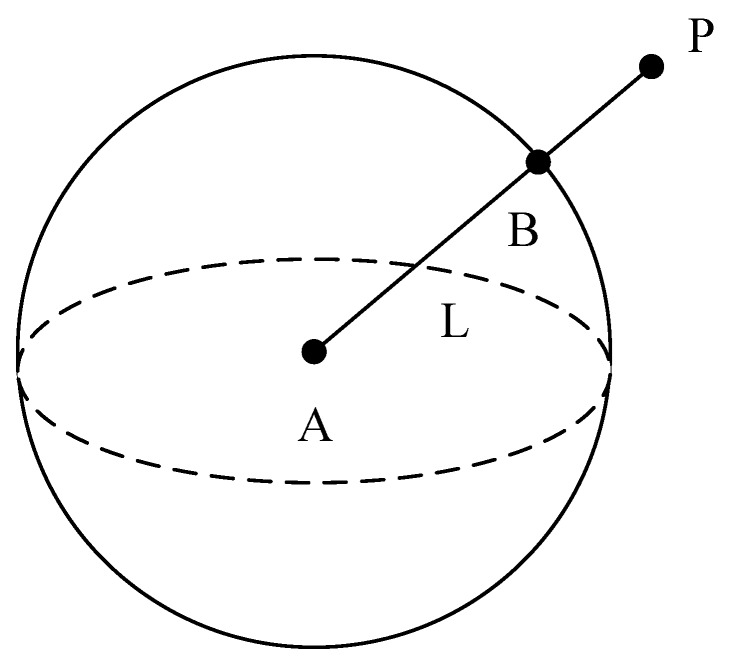
Schematic diagram of the position of error joint B.

Finally, the estimated joint position $\left(\tilde{X},\tilde{Y},\tilde{Z}\right)$ is optimized. By establishing a spatial linear equation between $P(\tilde{X},\tilde{Y},\tilde{Z})$ and A$\left(X_{1},Y_{1},Z_{1}\right)$, we can acquire optimized joint position B$\left(\hat{X},\hat{Y},\hat{Z}\right)$, which is on the constraint equation and closest to the estimated joint position $P(\tilde{X},\tilde{Y},\tilde{Z})$, as shown in Figure 3.

The constraint equation intersects the linear equation at two points. The solution with the smallest coordinate distance from the joint estimated position point *P* is selected as the final estimated error of the joint optimization estimated position.

(15) $$\hat{X}=\pm \sqrt{\frac{{l_{AB}}^{2}(\tilde{X}-X_{1})}{{\left(\tilde{X}-X_{1}\right)}^{2}+{\left(\tilde{Y}-Y_{1}\right)}^{2}+{\left(\tilde{Z}-Z_{1}\right)}^{2}}}+\tilde{X}$$

(16) $$\hat{Y}=\pm \sqrt{\frac{{l_{AB}}^{2}(\tilde{Y}-Y_{1})}{{\left(\tilde{X}-X_{1}\right)}^{2}+{\left(\tilde{Y}-Y_{1}\right)}^{2}+{\left(\tilde{Z}-Z_{1}\right)}^{2}}}+\tilde{Y}$$

(17) $$\hat{Z}=\pm \sqrt{\frac{{l_{AB}}^{2}(\tilde{Z}-Z_{1})}{{\left(\tilde{X}-X_{1}\right)}^{2}+{\left(\tilde{Y}-Y_{1}\right)}^{2}+{\left(\tilde{Z}-Z_{1}\right)}^{2}}}+\tilde{Z}$$

## 3. Experimental Setup

**Figure 4:**
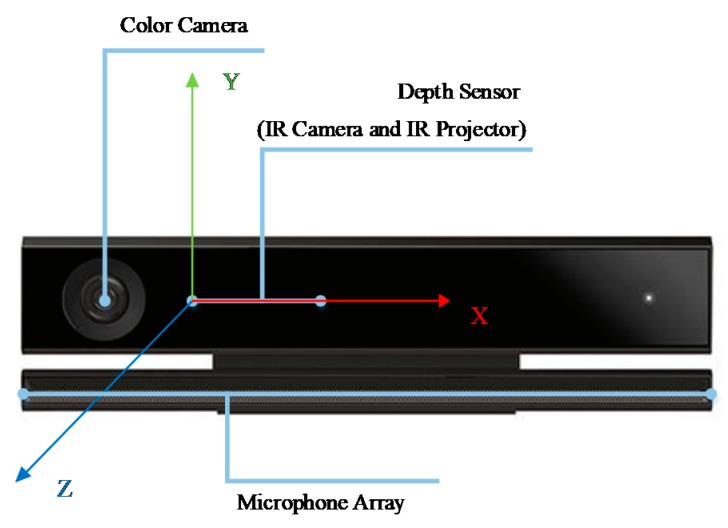
Kinect sensor and its coordinate system (IR means infrared).

Our experiment is based on Kinect version 2.0, which provides pose estimations for 25 “skeleton” joints at 30 Hz and enables the tracking of a user’s skeleton on a subset of joints [21]. A schematic of the Kinect, its sensor locations and its right-handed coordinate system is shown in Figure 4. The Kinect base sits parallel to the (x, z) plane, and the origin of the coordinate is at the center of the infrared camera. The X-axis runs parallel through the video and audio sensor arrays, the Y-axis runs perpendicular to the Kinect base, and the Z-axis defines the illumination direction. The coordinate unit is meter (m).

**Figure 5:**
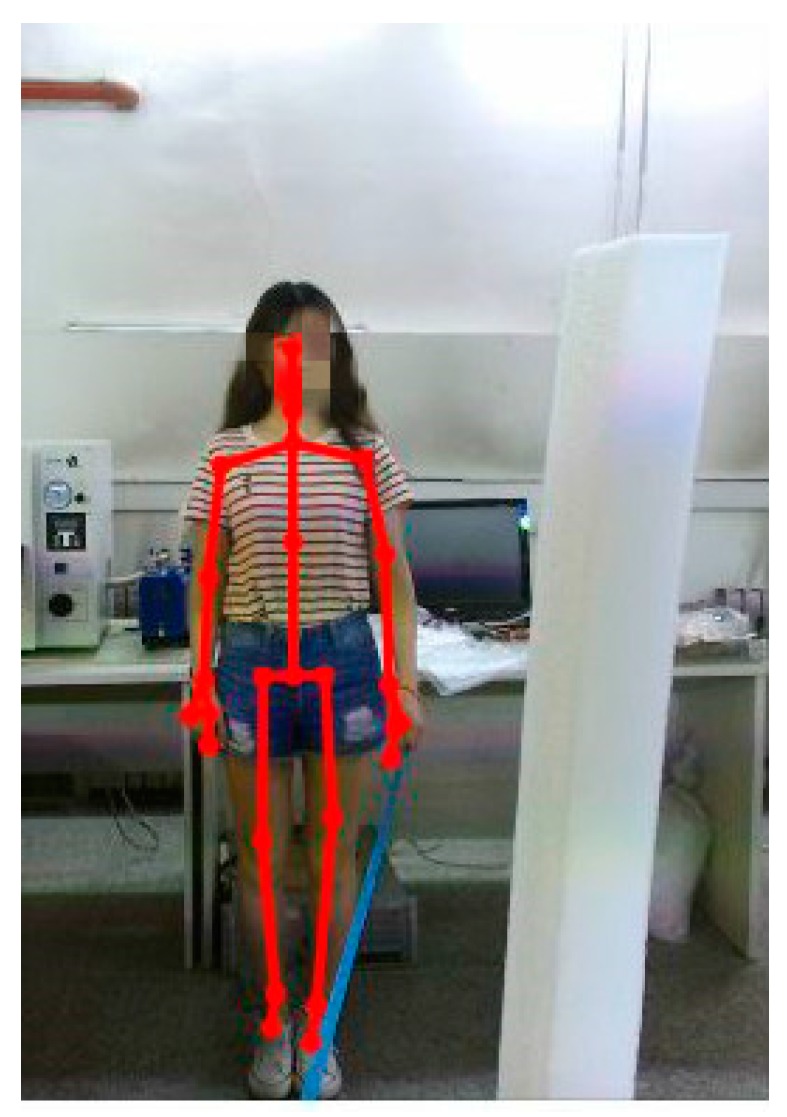
Occlusion experimental setup.

In general, the accuracy of Kinect is evaluated by comparing data collected by Kinect with data acquired by optical motion capture devices (such as VICON). However, as described in Section 2.2, all sensors produce a few measurement errors when measuring physical quantities. Thus, we may not be able to obtain the most accurate joint position trajectory. Since the precise trajectory is difficult to measure, this paper abandoned the use of an optical motion capture instrument to obtain human skeleton joint positions as the ground truth. Instead, we adopted the trajectory acquisition method presented in [19], which first set the fixed point on the ground as the center of the special motion trajectory in the (X, Z) plane. The present paper selected the quarter circular trajectory. First, we determined a point as the center of the quarter circular trajectory, which implies that we fixed the Y-direction coordinate of the human joint position. Then, we took a piece of a tape measure and attached it to the fixed point. Finally, we instructed the subject to face the Kinect at all times and move along the quarter circular path while holding the other end of the tape at the skeleton wrist joint. The obtained quarter circular trajectory of the wrist joint is considered to be the ground truth. Unlike [19], we added an obstruction to the joint trajectory to generate incorrect data. In this experiment, a total of five subjects’ upper limb movement data were collected, and the experiment was repeated five times for each subject with 120 frames of experimental data. The experimental scene is shown in Figure 5. 

## 4. Results and Discussion

**Figure 6:**
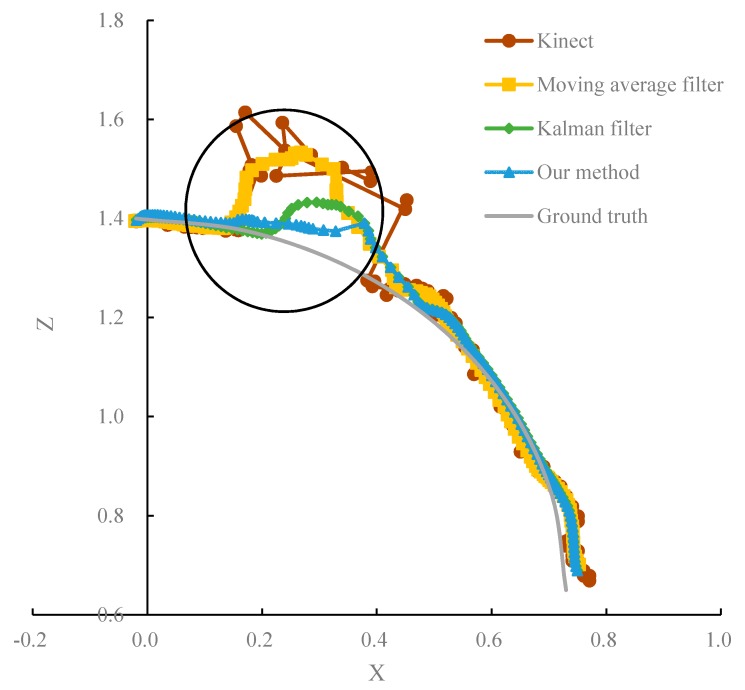
Accuracy comparison of different algorithms and the true trajectory.

Figure 6 below shows the performance of tracking the wrist trajectory using the original Kinect, the moving mean filter algorithm, the traditional Kalman filter algorithm and our method compared to the ground truth. The trajectory shown in the black circle in Figure 6 is erroneous data caused by occlusion. 

From Figure 6, it is observed that the algorithm proposed in this paper is superior to the other algorithms. The idea of our method is to separate erroneous data from noise data, perform targeted processing of the identified erroneous data, discard the original erroneous data and estimate the new joint position by combining the human constraint and filtering prediction as the current joint position. Therefore, the algorithm presented in this paper is less affected by external measurement data and maintains a similar trend to the real trajectory near the erroneous data. The ordinary filtering method applies the erroneous data to the smoothing process, which is greatly affected by the external measurement data. Therefore, the movement trend of the measurement data will remain in the vicinity of the erroneous data, and the deviation is large.

To measure accuracy, the average error of the estimated joint position and the true trajectory were calculated using the following formula:

(18) $$E=\frac{\sum_{i=1}^{n}\sqrt{{\left(x(i)-x_{0}(i)\right)}^{2}+{\left(z(i)-z_{0}(i)\right)}^{2}}}{n}$$

where x(i) and z(i) are the x and z components of the human joint position coordinate of the *i* th frame processed by different algorithms, respectively; $x_{0}\left(i\right)$ and $z_{0}\left(i\right)$ are the x and z components of the human joint position coordinate of the *i* th frame in the true trajectory, respectively. 

Table 2 shows the error of the original joint movement trajectory acquired by Kinect; the joint trajectories processed by the moving mean filter algorithm, the traditional Kalman filter algorithm and the algorithm proposed in this paper; and the true geometric trajectory. Table 2 shows that the joint data processing algorithm proposed in this paper is superior to the other algorithms in regard to the overall average error comparison. Based on the original Kinect data, the data accuracy was improved by 21.3% after moving mean filter. After the traditional Kalman filter processing, the data accuracy is increased by 46.7%, and after the algorithm proposed in this paper processing, the data accuracy is increased by 58.7%.

As for computational efficiency, though the algorithm complexity of our method is higher than other algorithm like moving mean filter algorithm, it is not obvious in terms of the difference. Since the moving mean filter algorithm is simple, we observed that it takes roughly 0.975 s to process one experiment sample, whereas it takes roughly 1.248 s for traditional Kalman filter to process one sample. Our proposed method adds classification algorithm before extend Kalman filter so that it takes roughly 1.592 s to adapt one sample. All the algorithms are performed on the MATLAB 2016b platform with 3.1 GHz Intel Core i5 Processor.

## 5. Conclusions and Future Work

Regarding the accuracy of Kinect, few studies have focused on improving the inherent skeleton tracking accuracy of Kinect. These studies simply intended to show that applications based on Kinect could be significantly improved by applying optimal techniques. In this process, the researchers ignored the generalization of methods to improve the accuracy of Kinect. This tendency is susceptible to the embarrassing situation that the method is suitable for posture assessment but not home rehabilitation. We proposed a novel algorithm to improve the accuracy of Kinect skeletal joint coordinates for improving the inherent accuracy of Kinect. Our method introduced a skeletal joint data classification algorithm to divide noise data and erroneous data. Furthermore, we proposed two different algorithms to smooth noise data and correct erroneous data to accurately the track dynamic trajectory joint center location over time. Our method can potentially expand the way to process Kinect data in applications based on Kinect because we separate Kinect data processing from applications. Thus, our method is suitable for most applications related to Kinect. 

The present paper evaluated the algorithm in an occlusion experiment. The results of these experiments are significant. The results show that the algorithm substantially smooths the skeleton joint position estimates of Kinect; more importantly, the experiments demonstrate that the tracking accuracy is significantly increased. In this study, we compared the results of our method with the original Kinect data, the moving mean filter algorithm and the traditional Kalman filter algorithm and obtained an accuracy improvement of 58.7%, 47.5% and 22.5%, respectively. As a result, using the skeletal joint data classification algorithm and two different data-processing algorithms to smooth noise data and correct erroneous data reduce the average estimation error for tracking human dynamic skeleton joints.

However, there are limitations to this study. Our proposed algorithm for Kinect skeletal joint data classification only considers the vibration between frames. However, there is also a limited relationship between the coordinates of each joint point in the same frame. For future work, we plan to enrich the skeletal joint data classification algorithm by incorporating the limit relationship. Furthermore, the setting of the values like reliability threshold is a shortcoming. We should consult with expertise from physiology, rehabilitation or even neuroscience to determine the reference threshold, and we should have conducted some preliminary experiments to verify the rationality of the data difference. In addition, we only considered the tracking of the (*x*, *z*) coordinate of the wrist joint of the subject with a known quarter circular trajectory. The results must be verified based on more complex motions. However, the true trajectories are difficult to measure and may require more sophisticated and expensive equipment, which will be conducted in possible future research.

## Figures and Tables

**Table 1:** Reliability threshold results.

Experiment	Reliability Threshold				
	Subject 1	Subject 2	Subject 3	Subject 4	Subject 5
1	0.69	0.68	0.74	0.72	0.79
2	0.70	0.73	0.65	0.77	0.75
3	0.72	0.69	0.64	0.76	0.66
4	0.75	0.74	0.63	0.73	0.73
5	0.66	0.65	0.66	0.69	0.69

**Table 2:** Comparison of the error of different algorithms (unit: m).

	Kinect		Moving Mean Filter		Kalman Filter		Our Method	
	Error	SD	Error	SD	Error	SD	Error	SD
1	0.081	0.008	0.065	0.007	0.043	0.003	0.032	0.003
2	0.076	0.004	0.061	0.005	0.041	0.002	0.031	0.003
3	0.071	0.004	0.054	0.010	0.036	0.003	0.028	0.005
4	0.069	0.007	0.051	0.009	0.039	0.002	0.030	0.002
5	0.078	0.005	0.062	0.004	0.042	0.004	0.036	0.004
Mean	0.075	0.006	0.059	0.007	0.040	0.003	0.031	0.003

SD=Standard Deviation.

## Data Availability

The data used to support the findings of this study are included within this paper. It is also available from the corresponding author upon request.
